# Anti-human TLR7 antibody for therapeutic intervention in systemic lupus erythematosus

**DOI:** 10.1093/intimm/dxaf046

**Published:** 2025-09-02

**Authors:** Ryutaro Fukui, Yusuke Murakami, Atsuo Kanno, Yuji Motoi, Atsushi Manno, Tomohiro Honda, Shinnosuke Yamada, Jun Ishiguro, Takashi Kagari, Kensuke Nakamura, Michinori Kadokura, Takashi Isobe, Yoshiaki Tomimori, Jun Tanaka, Giorgio Senaldi, Toshiyuki Shimizu, Kensuke Miyake

**Affiliations:** Division of Innate Immunity, Department of Microbiology and Immunology, The Institute of Medical Science, The University of Tokyo, Tokyo, Japan; Synergy Institute for Futuristic Mucosal Vaccine Research and Development, Chiba University, Chiba, Japan; Medical Mycology Research Center, Chiba University, Chiba, Japan; Division of Innate Immunity, Department of Microbiology and Immunology, The Institute of Medical Science, The University of Tokyo, Tokyo, Japan; Faculty of Pharmacy, Department of Pharmaceutical Sciences, Research Institute of Pharmaceutical Sciences, Musashino University, Tokyo, Japan; Division of Innate Immunity, Department of Microbiology and Immunology, The Institute of Medical Science, The University of Tokyo, Tokyo, Japan; Division of Innate Immunity, Department of Microbiology and Immunology, The Institute of Medical Science, The University of Tokyo, Tokyo, Japan; Synergy Institute for Futuristic Mucosal Vaccine Research and Development, Chiba University, Chiba, Japan; Discovery Research Laboratories II, Daiichi Sankyo Co., Ltd., Tokyo, Japan; Translational Science Department II, Daiichi Sankyo Co., Ltd., Tokyo, Japan; Translational Science Department II, Daiichi Sankyo Co., Ltd., Tokyo, Japan; Discovery Research Laboratories V, Daiichi Sankyo Co., Ltd., Tokyo, Japan; Discovery Research Laboratories I, Daiichi Sankyo Co., Ltd., Tokyo, Japan; Modality Research Laboratories II, Daiichi Sankyo Co., Ltd., Tokyo, Japan; Modality Research Laboratories II, Daiichi Sankyo Co., Ltd., Tokyo, Japan; Translational Research Laboratories, Daiichi Sankyo Co., Ltd., Tokyo, Japan; Translational Science, Daiichi Sankyo, Inc., Basking Ridge, NJ, USA; Translational Science Department II, Daiichi Sankyo Co., Ltd., Tokyo, Japan; Clinical Development, Daiichi Sankyo, Inc., Basking Ridge, NJ, USA; Graduate School of Pharmaceutical Sciences, The University of Tokyo, Tokyo, Japan; Division of Innate Immunity, Department of Microbiology and Immunology, The Institute of Medical Science, The University of Tokyo, Tokyo, Japan; Synergy Institute for Futuristic Mucosal Vaccine Research and Development, Chiba University, Chiba, Japan; Medical Mycology Research Center, Chiba University, Chiba, Japan

**Keywords:** antibody drug, autoimmunity, gene signature, humanized antibody

## Abstract

Toll-like receptor 7 (TLR7) is an endosomal sensor that responds to both pathogen-derived and self-derived single-stranded RNA (ssRNA). Responses of TLR7 to self-derived ssRNA have been implicated in the development of autoimmune diseases, such as systemic lupus erythematosus (SLE). TLR7 antagonists and inhibitory anti-TLR7 monoclonal antibodies (mAbs) can protect lupus-prone NZBWF1 mice from lethal nephritis. However, less is known about TLR7 dependence and activation in human SLE, as both TLR7 and TLR8 respond to ssRNA in humans. Here, we analyzed public databases and found that TLR7 gene signature scores were consistently elevated across datasets, races, and SLEDAI scores compared to TLR8, suggesting a deeper involvement of TLR7 in SLE pathogenesis. To specifically inhibit human TLR7 responses, we developed inhibitory mAbs against human TLR7. Utilizing an inhibitory clone, we generated the humanized mAb, DS-7011a. DS-7011a effectively inhibited TLR7-mediated responses in plasmacytoid dendritic cells (pDCs) and B cells. Furthermore, DS-7011a was internalized in a TLR7-dependent manner and accumulated in B cells, pDCs, conventional dendritic cells (cDCs), and monocytes/macrophages. In this study, we describe the generation and preclinical development of DS-7011a, which has the potential to be a therapeutic option for the treatment of SLE.

## Graphical Abstract

**Graphical Abstract:**
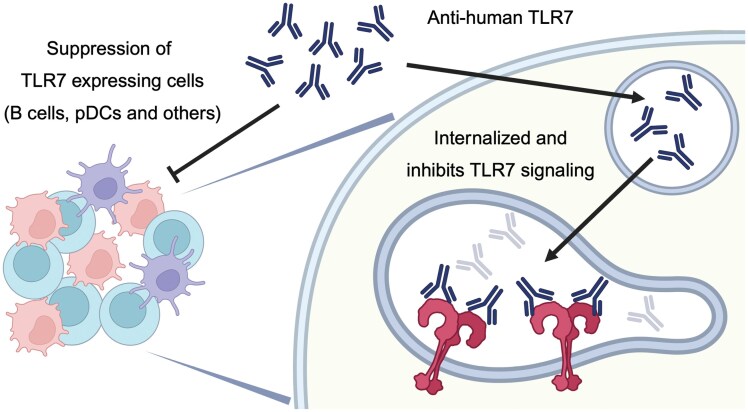

## Teaser

An anti-human TLR7 mAb that may ameliorate SLE

## Introduction

Systemic lupus erythematosus (SLE) is a multifaceted autoimmune disease with an unknown etiology, characterized by the production of autoantibodies and a diverse range of clinical manifestations affecting the skin, joints, kidneys, and central nervous system (1). Current therapeutic approaches mainly involve immunosuppressive agents such as the antimalarial drug hydroxychloroquine (HCQ), glucocorticoids, methotrexate (MTX), and mycophenolate mofetil (MMF). Despite these treatments, a number of patients develop severe and life-threatening conditions like lupus nephritis. Additionally, the long-term use of glucocorticoids is linked to numerous adverse effects, which limit their clinical utility (2). Hence, there is a pressing need to develop novel therapeutic agents that offer effective disease management with a reduced risk of adverse effects.

Toll-like receptor 7 (TLR7) and TLR8 are innate immune sensors for single-stranded RNA (ssRNA). These receptors recognize not only pathogen-derived ssRNAs but also self-derived ssRNAs. Hyper-responsiveness of TLR7 has been implicated in the development of murine models of autoimmune diseases such as SLE and psoriasis (3–5). The lupus-prone mouse strain, Y-linked autoimmune accelerator (Yaa), carries an extra copy of the TLR7 gene, leading to TLR7 hyperactivation (6, 7). Moreover, topical application of the TLR7 agonist imiquimod induces lupus nephritis in mice (8, 9). In humans, gain-of-function mutations of TLR7 result in lupus-like monogenic diseases (10). Furthermore, enhanced TLR7 responsiveness has been observed in plasmacytoid dendritic cells (pDCs) from patients with SLE, suggesting that TLR7 contributes to the pathogenesis of disease (11, 12). Conversely, gain-of-function mutations of TLR8 causes inborn errors of immunity characterized by neutropenia, antibody deficiency, and lymphoproliferation (13). Despite these studies, the role of TLR7 and TLR8 in pathogenesis of SLE has not been fully understood.

In preclinical models, inhibition of TLR7 or dual inhibition of TLR7/8 has been shown to ameliorate lupus nephritis in lupus-prone strains such as the New Zealand Black/New Zealand White F1 mice (NZBWF1 mice) and MRL/lpr mice (14–17). Furthermore, studies in humans indicate that blocking TLR7 and TLR8 can reduce IFNα production by peripheral blood mononuclear cells (PBMCs) stimulated with SLE immune complexes (15, 18). From these results, small molecules targeting both receptors are under clinical investigation (15, 18). However, these small molecules do not specifically inhibit TLR7-mediated signaling pathways. We previously developed an anti-mouse TLR7 monoclonal antibody (mAb), and this antibody inhibits TLR7-mediated responses in B cells, dendritic cells (DCs), and monocyte/macrophages (19). Although TLR7 is located in the endosomal compartment, this anti-mouse TLR7 mAb is internalized and binds to endosomal TLR7 (19). Moreover, this anti-TLR7 mAb ameliorates lupus nephritis in NZBWF1 mice (19, 20). These findings suggest that anti-TLR7 mAb is a promising tool for highly specific intervention in TLR7-mediated signaling pathways.

In this study, we investigated the TLR7 and TLR8 gene signature (GS) expression in SLE patients. For the treatment of SLE, we generated anti-human TLR7 (HuTLR7) mAbs and developed a humanized IgG1 mAb, DS-7011a. DS-7011a effectively inhibits TLR7-dependent IFNα production in pDCs and IgG production in B cells *in vitro*. These findings suggest that DS-7011a holds significant promise as a therapeutic option for SLE.

## Methods

### Reagents and antibodies

R848 and CL264 were purchased from InvivoGen (San Diego, CA, USA). CpG-B 1668 (5’-TCCATGACGTTCCTGATGCT-3’, whole phosphorothioated), CpG-B 2006 (5’-TCGTCGTTTTGTCGTTTTGTCGTT-3’, whole phosphorothioated), and ssRNA9.2s (5’-UGUCCUUCAAUGUCCUUCAA-3’, whole phosphorothioated) were synthesized by FASMAC (Atsugi, Japan). CpG-K3 was provided by Prof. Ishii (The University of Tokyo). Lipid A Re595 and DOTAP were purchased from Sigma-Aldrich (St. Louis, MO, USA). Recombinant mouse GM-CSF and human IFNγ were purchased from PeproTech (Rocky Hill, NJ, USA). Recombinant human soluble CD40L was purchased from ENZO Life Sciences (Farmingdale, NY, USA).

Anti-human TLR7, anti-human TLR8, anti-mouse TLR7, anti-human TLR4, and anti-BFP (blue fluorescent protein) were prepared by us. The anti-human TLR4 (clone TF904) and anti-BFP (clone B321) were used as mouse IgG1 isotype controls. Humanized anti-human TLR7 mAb (DS-7011a) and isotype control were prepared by Daiichi Sankyo Co., Ltd.

Anti-Mouse IgG, anti-mouse IgG1, streptavidin (StAv), anti-DYKDDDDK, anti-mouse CD3, anti-mouse CD19, anti-mouse B220, anti-mouse CD21, anti-mouse CD23, anti-mouse CD11c, anti-mouse I-A/I-E, anti-mouse Ly6C, anti-mouse Ly6G, anti-mouse Siglec-H, anti-human CD19, anti-human CD4, anti-HLA-DR, anti-human CD11c, anti-human CD1c, and anti-human CD16 were purchased from BioLegend (San Diego, CA, USA). Anti-mouse CD49b, anti-mouse/human CD11b, anti-mouse Ly6G, anti-mouse CD16.2, anti-mouse Siglec-H, anti-mouse PDCA1, anti-human CD3, anti-human CD123, anti-human CD14, and anti-human CD15 were purchased from Becton Dickinson (Franklin Lakes, NJ, USA). Anti-Alexa Fluor 488 and anti-human CD16 were purchased from Thermo Scientific (Waltham, MA, USA). Anti-mouse CD16/32 was purchased from Bio X cell (Lebanon, NH, USA).

### Cell culture

We prepared four types of complete RPMI 1640 medium. Type A is RPMI 1640 (Nacalai, Kyoto, Japan) supplemented with 10% fetal bovine serum (FBS) (Sigma), 1x penicillin-streptomycin-glutamine (PS/Gln, Nacalai), and 50 μM 2-ME (Nacalai). Type B is RPMI 1640 medium (Thermo Fisher Scientific, Inc., Waltham, MA, USA) supplemented with 10% FBS (Mediatech, Inc., Herndon, VA, USA), 1% sodium pyruvate (100 mM) (Thermo Fisher Scientific, Inc.), 1% MEM nonessential amino acids solution (100x) (Thermo Fisher Scientific, Inc.), 0.1% 2-ME (Thermo Fisher Scientific, Inc.), and 1% penicillin-streptomycin (Thermo Fisher Scientific, Inc.). Type C is RPMI 1640 medium (Invitrogen, Thermo Scientific, Inc.) supplemented with 10% FBS (MP Biomedicals, Santa Ana, CA, USA), 1% sodium pyruvate (100 mM) (Invitrogen, Thermo Scientific, Inc.), 1% MEM nonessential amino acids solution (100x) (Invitrogen, Thermo Scientific, Inc.), 0.1% 2-ME (Invitrogen, Thermo Scientific, Inc.), and 1% penicillin-streptomycin (Invitrogen, Thermo Scientific, Inc.). Type D is RPMI 1640 (Invitrogen, Thermo Scientific, Inc.) supplemented with 10% FBS (MP Biomedicals), 1% penicillin-streptomycin (Thermo Fisher Scientific, Inc.).

Ramos cells, hybridomas, and conventional DCs (cDCs) were cultured in type A medium. Frozen human PBMCs were cultured in type A (Figs 4E and 5H) or type B (Fig. 5D). Fresh human PBMCs were cultured in type C. Frozen human pDCs and B cells were cultured in type D medium.

**Figure 1.**
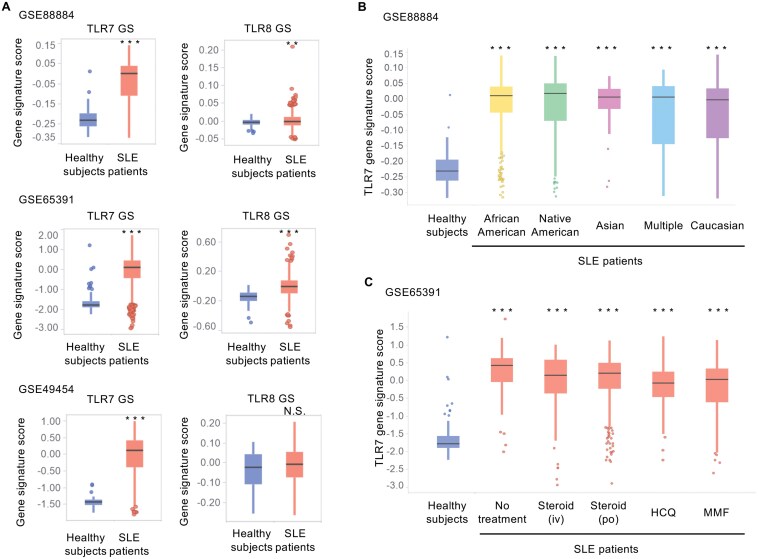
TLR7 gene signature scores are enhanced in SLE patients and their subgroups. (A) Comparison of SLE patients and healthy subjects for TLR7 and TLR8 gene signature scores in blood samples (GSE88884, SLE, *N* = 1760; healthy, *N* = 60. GSE65391, SLE, *N* = 924; healthy, *N* = 72. GSE49454, SLE, *N* = 157; healthy, *N* = 20). (B) Ethnic differences of TLR7 gene signature scores (African American, *N* = 233; Native American, *N* = 249; Asian, *N* = 19; Multiple, *N* = 39; Caucasian, *N* = 1215) (GSE88884). (C) TLR7 gene signature scores of SLE patients with or without drug treatment (No treatment, *N* = 40; steroid (iv), *N* = 86; steroid (po), *N* = 331; HCQ, *N* = 190; MMF, *N* = 358) and healthy subjects (*N* = 72) in blood samples (GSE65391). Data were statistically analyzed by Welch’s two-sample *t*-test (A), Dunnett’s multiple comparison test (B), and Tukey’s multiple comparison test (C). N.S., not significant; **, *P* < .01; ***, *P* < .001. See also Supplementary Figs 1, 2, and Supplementary Table 1.

**Figure 2.**
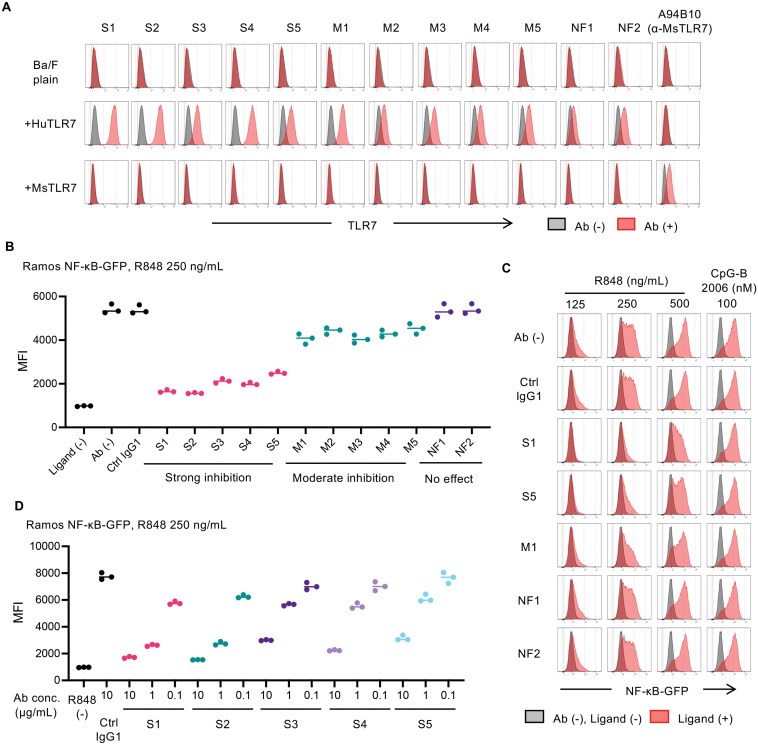
Development of anti-human TLR7 monoclonal antibodies. (A) Staining pattern of anti-HuTLR7 monoclonal antibodies (mAbs). Ba/F3 cells were fixed and permeabilized for intracellular staining of HuTLR7-FH (FLAG-6xHis) and mouse TLR7 (MsTLR7)-FH. (B) NF-κB-GFP reporter assay using the B-cell lymphoma cell line Ramos. Cells were treated by 10 μg/mL of mAbs for 4 hours and stimulated with the indicated ligands. 22 hours after stimulation, the expression of GFP was measured with flow cytometry to detect the activation of NF-κB. (C) Representative histograms of (B). (D) Ramos cells were treated with the indicated mAbs at the indicated concentrations for 4 hours and stimulated with 250 ng/mL of R848. The expression of NF-κB-GFP was measured by flow cytometry. At least three independent experiments were performed (A-D). Data are shown as individual points and as means for each experimental group (B and D). See also Supplementary Fig. 3.

**Figure 3.**
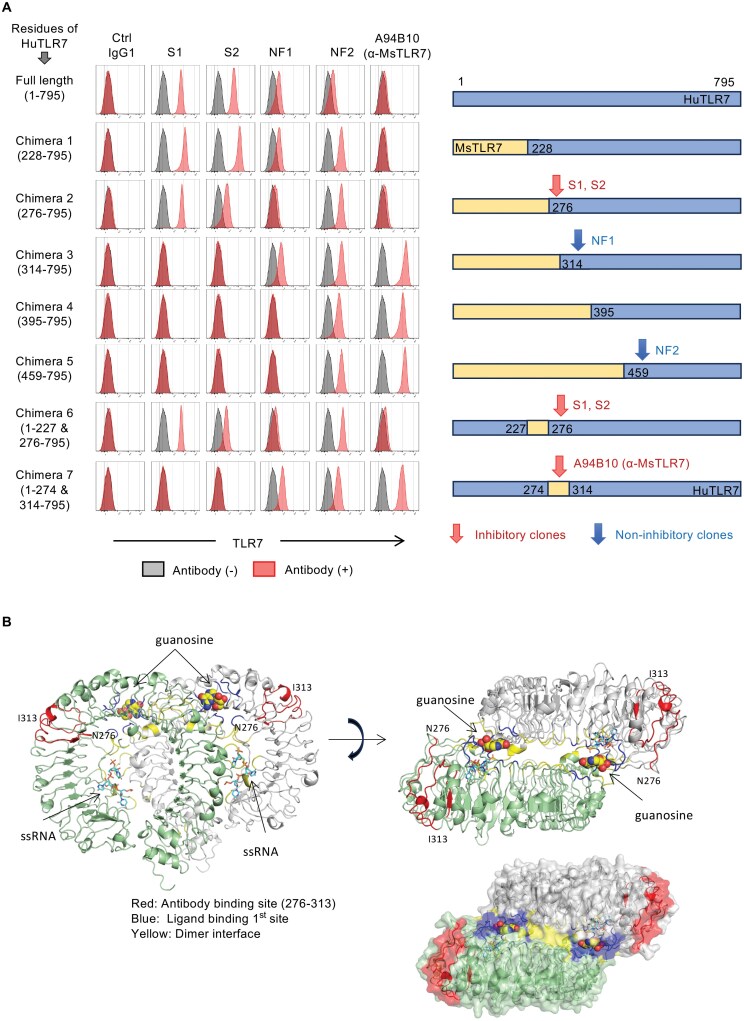
Inhibitory clones of anti-humanTLR7 mAb recognize the N-terminal region of TLR7. (A) Membrane-permeabilized staining of Ba/F3 cells expressing human/mouse chimeric TLR7 proteins with indicated clones. Configurations of chimeric proteins are shown to the right. The transmembrane region and TIR domain were not shown. (B) Schematic representation of the structure of monkey TLR7 (PDB ID: 5GMF). The amino acid sequence of the epitope of clone S1 is the same in human TLR7 and monkey TLR7. Front (left) and top (upper right) views, and molecular surface (lower right) are shown. TLR7 and its dimerization partner are colored green and gray. The red regions from N276 to I313 show the epitope of clone S1. The blue and yellow regions show nucleoside-binding sites and dimerization interfaces, respectively. The C, O, N, and P atoms of the ligands are colored yellow (uridine) or cyan (ssRNA), red, blue, and orange, respectively. At least 3 independent experiments were performed, and representative data were shown (A).

**Figure 4.**
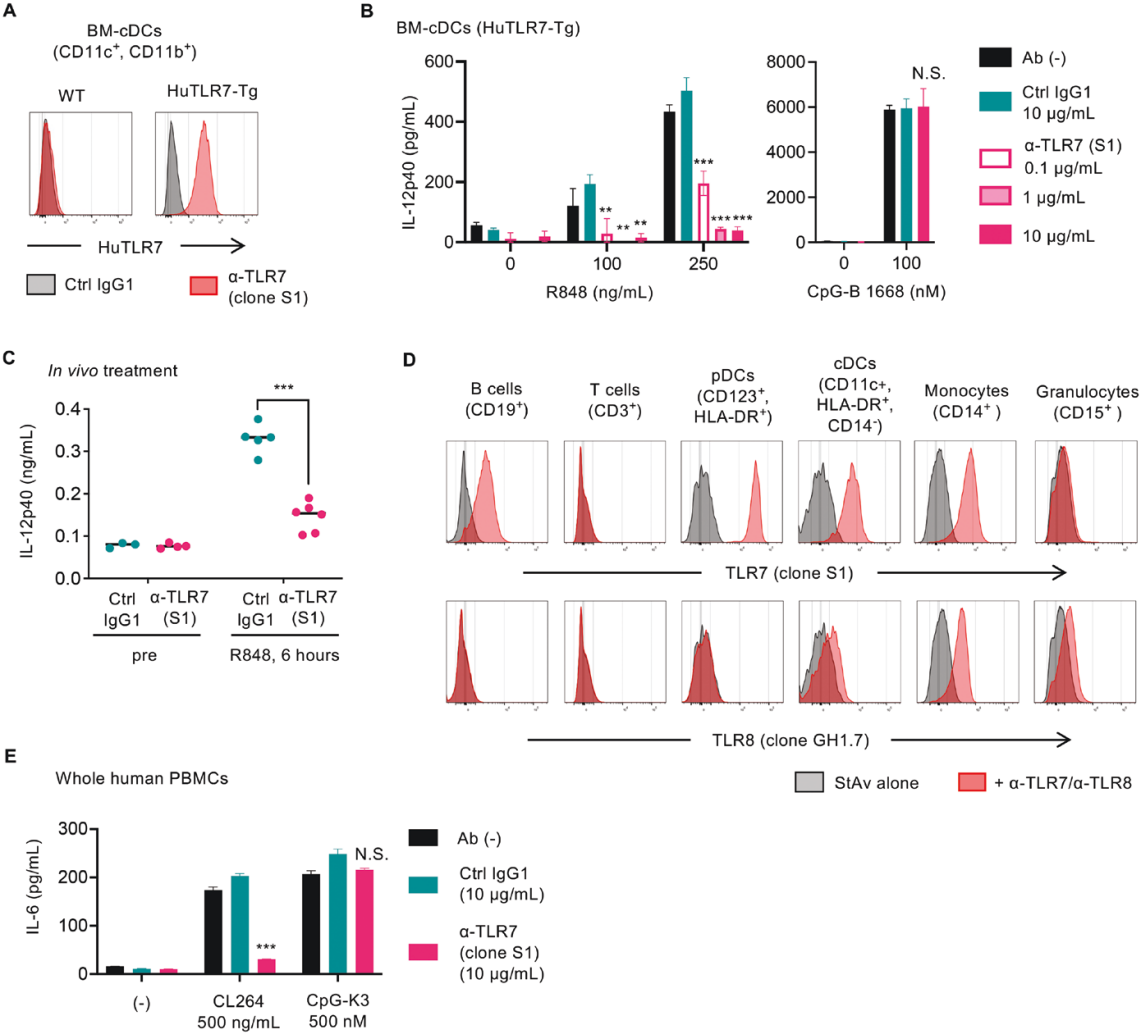
Anti-human TLR7 mAb inhibits the response of TLR7 in human PBMCs and the human TLR7 transgenic mice. (A) Human TLR7 expression in the bone marrow (BM)-derived cDCs from WT or HuTLR7 transgenic (Tg) mice. Cells were fixed and permeabilized for the detection of HuTLR7. (B) Inhibition of TLR7 response in the BM-cDCs derived from HuTLR7-Tg mice. After 4 hours of incubation with Abs, cells were stimulated by TLR7 or TLR9 ligand. Cells were incubated for 24 hours, and the concentrations of IL-12 p40 in the culture medium were measured by ELISA. (C) The inhibitory effect of clone S1 on TLR7 response in HuTLR7-Tg mice. 25 mg/kg of anti-HuTLR7 mAb (clone S1) and control IgG1 (clone TF904) were administrated to WT (*N* = 5) and HuTLR7-Tg mice (*N* = 6). Seven days after Ab administration, 20 μg of R848 was intraperitoneally administered. Six hours after R848 injection, blood was collected and the serum levels of IL-12 p40 were determined by ELISA. (D) TLR7 and TLR8 expression in human PBMCs. After staining with mAbs to indicated lineage markers, the cells were fixed and intracellular TLR7 or TLR8 was stained by clone S1 or anti-HuTLR8 (clone GH1.7), respectively. (E) Inhibition of TLR7 responses by anti-TLR7 mAb. Human PBMCs were treated with mAbs for 4 hours, and then stimulated with TLR7 ligand (CL264) or TLR9 ligand (CpG-K3). 24 hours after stimulation, culture supernatants were collected and concentrations of IL-6 were measured by ELISA. At least 3 independent experiments were performed (A-E), and representative histograms were shown (A and D). Wells of ELISA were triplicated, and the mean SD was shown (B and E). Dots in the graph indicate the data from individual mice (C). PBMCs were collected from at least 3 healthy donors (D and E). Data were statistically analyzed by one-way ANOVA with multiple comparisons (B and E) or Student’s T-test (C). N.S., not significant; ** *P* < 0.01; *** *P* < .001. See also Supplementary Fig. 4.

**Figure 5.**
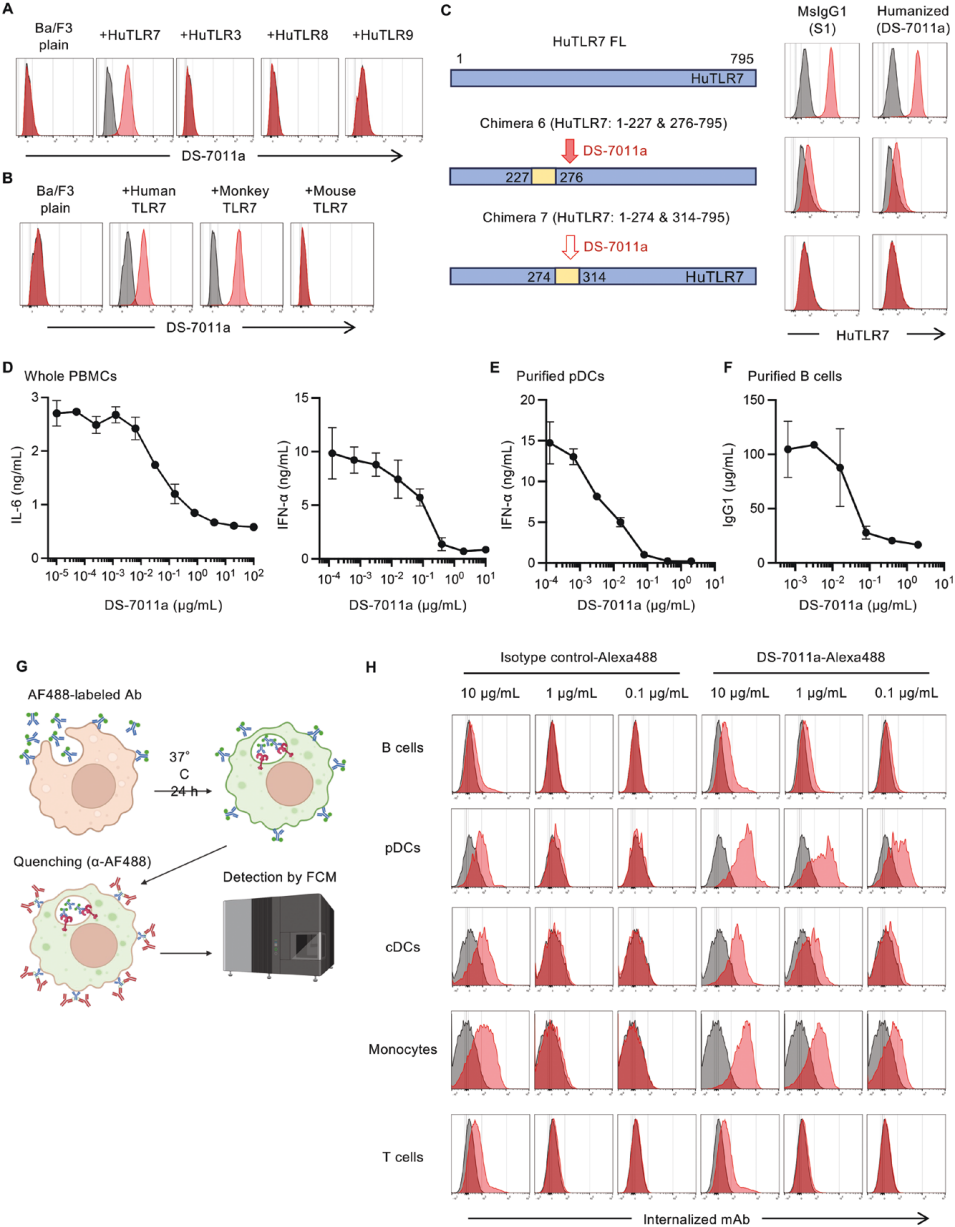
Humanized anti-TLR7 mAb, DS-7011a, is internalized into cells and inhibits the TLR7 response. (A–C) Intracellular staining of Ba/F3 cells expressing indicated TLRs. Gray histograms and red histograms show staining with the isotype control and DS-7011a, respectively. Alexa fluor 488 (AF488)-labeled antibodies were used for (C). (D) Inhibitory effect of DS-7011a on IL-6 production by CL264 stimulation and IFN-α production by ssRNA9.2s stimulation in human PBMCs. TLR7-dependent cytokine production by DS-7011a. Whole human PBMCs were incubated with DS-7011a for 4 hours before stimulation with CL264 (1 μg/mL) or ssRNA9.2s (1 μg/mL) as a TLR7 ligand, respectively. Twenty hours after stimulation, culture supernatants were collected and concentrations of IL-6 and IFN-α were measured by AlphaLISA. (E) Inhibitory effect of DS-7011a on IFN-α production by ssRNA9.2s stimulation in purified human pDCs. Purified human pDCs were incubated with DS-7011a for 4 hours before stimulation with ssRNA9.2s (1 μg/mL) as a TLR7 ligand. Twenty hours after stimulation, culture supernatants were collected and concentration of IFN-α was measured by AlphaLISA. (F) Inhibition of TLR7-dependent IgG1 production by DS-7011a. Purified human B cells were incubated with DS-7011a for 4 hours before stimulation with CL264. Five days after stimulation, culture supernatants of B cells were collected, and concentrations of IgG1 were measured by cell-based ELISA. (G) Illustration of the method for antibody uptake assay. (H) Internalization of DS-7011a in human PBMCs. AF488-labeled humanized anti-TLR7 mAb (DS-7011a) and isotype control mAb were incubated with PBMCs for 24 hours at 37°C. Antibody on the cell surface was quenched by anti-AF488, and the fluorescence of internalized antibody was detected by flow cytometry. Gray histograms show the data without antibody. Red histograms show the data with AF488-labeled antibodies. At least three independent experiments were performed, and representative histograms were shown (A–C, and H). PBMCs, purified pDCs, and purified B cells were collected from at least 2 healthy donors (A–F, H, and Supplementary Fig. 5). Wells of AlphaLISA or cell-based ELISA were triplicated, and the mean SD was shown (D–F). See also Supplementary Fig. 5.

Ba/F3 cells were cultured in the complete RPMI 1640 medium supplemented with 1 ng/mL of recombinant murine IL-3 (Peprotech). To induce cDCs, bone marrow (BM) cells were plated at 1 × 10^7^ cells per 10 mL with complete RPMI 1640 medium and 20 ng/mL of recombinant GM-CSF for 7 days. Culture suspension of BM-cDCs was split every 2–3 days. PLAT-E retrovirus packaging cells were cultured in DMEM (Nacalai) supplemented with 10% FBS, 1x PS/Gln, 50 μM 2-ME, 10 μg/mL of blasticidine (InvivoGen), and 1 μg/mL of puromycin (InvivoGen) on a collagen-coated culture dish.

### Mice

Wild-type BALB/c and C57BL/6N mice were purchased from Japan SLC (Hamamatsu, Japan). BALB/c background *Tlr9*^−/−^ mice were generated from C57BL/6N background *Tlr9*^−/−^ mice by over 15 times of backcrossing (21). We previously established *Rosa26*^huTLR7/+^ mice where TLR7 mRNA is expressed under the Rosa26 promoter (22). These mice were kept in SPF conditions. All animal experiments were approved by the Animal Research Committee of The Institute of Medical Science, The University of Tokyo (IMSUT), and performed in accordance with guidelines.

### Gene signature analysis

Three whole blood transcriptome datasets from SLE patients were obtained from the Gene Expression Omnibus database. The TLR7 and TLR8 gene signatures (GSs) were defined based on the experimental data (Supplementary Table 1). The strength scores were calculated as GS scores (23), compared between healthy subjects and SLE patients, or subgroups of SLE patients, for the evaluation of relationships.

### Plasmid construction

Human TLR7, TLR3, TLR8, TLR9, and UNC93B1 were amplified by PCR and cloned into a retroviral pMXs vector. Mouse TLR7 and Monkey TLR7 were also cloned by the same protocol. FLAG-6xHis (FH) tag or HA tag was added as necessary. Prime STAR DNA polymerase series (TaKaRa Bio, Kusatsu, Japan) was used for PCR. The In-Fusion HD cloning kit (TaKaRa Bio) and Rapid DNA Ligation kit (Roche Applied Science) were used for cloning. pMX series was kindly provided by Dr Kitamura (IMSUT) (24).

### Retroviral transduction

Plasmids were transfected into PLAT-E packaging cells with FuGene6 (Promega, Madison, WI, USA). After 2 days of incubation, supernatants were collected as virus suspensions. Ba/F3 cells were transduced by virus suspensions mixed with DOTAP and centrifuged at 2000 rpm for 1 hour. Transgene expression was confirmed at least 3 days after transduction.

### Establishment of anti-humanTLR7 mAbs

FLAG-6xHis (FH)-tagged HuTLR7 and HA-tagged HuUnc93B1 (WT) genes were expressed in Ba/F3 cells. Over 5 × 10^7^ cells were collected and stimulated by 250 ng/mL of R848 for 2 hours at 37°C (cell-based antigen). Stimulated cells were washed in PBS 2 times and homogenized with TiterMax Gold (TiterMax, Norcross, GA, USA). For the initial immunization and the first boost, we injected the antigen into the foot pad, tail base, and peritoneal cavity (IP) of BALB/c background *Tlr9*^-/-^ mice. 240 μg of recombinant HuTLR7 protein was mixed with TiterMax Gold and used for the second IP boost. Four days before sacrifice, cell-based antigen was mixed with PBS and injected IP. Spleen and lymph nodes (inguinal, brachial, axillary, and retroperitoneal) were collected from immunized mice and suspended into single cells. The immunized cells and Sp2/O-Ag14 myeloma cells were mixed and fused by polyethylene glycol 1500 (PEG1500) to generate hybridoma. Cells were cultured in 96-well flat-bottom plates and selected by HAT supplement (GIBCO, Thermo Scientific). Cells were cultured until colonies were observable and subjected to screening by flow cytometry. The hybridomas in the positive wells were made into single cells by limiting dilution, and clones were obtained (Supplementary Fig. 3A).

### Establishment of anti-humanTLR8 mAb

WT BALB/C mice were immunized several times with purified HuTLR8 ectodomain and Ba/F3 cells expressing HuTLR8 mixed with TiterMax Gold. 4 days after the final immunization, splenocytes and SP2/O myeloma cells were mixed and fused with PEG1500. After selection by HAT supplement, antibodies against TLR8 were selected by flow cytometry using Ba/F3 cells expressing HuTLR8.

Anti-HuTLR8 was designated as GH1.7 (Supplementary Fig. 3C).

### Purification and labeling of mAbs

Hybridomas were acclimatized to serum-free medium (FUJIFILM Wako Pure Chemical Corporation, Osaka, Japan). Culture supernatant of hybridoma was collected and purified with a HiTrap Protein G HP Column and ÄKTA prime plus (Cytiva, Tokyo, Japan). The extracted antibody solution was subjected to buffer replacement with PBS using a PD-10 column (Cytiva). For antibody labeling, Biotin Labeling Kit—NH_2_ (Dojindo, Kumamoto, Japan) and Alexa Fluor 488 SiteClick sDIBO Alkyne Kits for Antibody Labeling (Invitrogen, Thermo Scientific) were used.

### Staining of cells

Staining buffer was prepared with 1xPBS, 2% FBS, 0.04% NaHCO_3_, 2 mM EDTA, and 0.1% NaN_3_. Cells were incubated with fluorescently conjugated antibodies for 15 minutes at 4°C. For intracellular staining of TLRs, cells were fixed and permeabilized by Cyto-Fast Fix/Perm Buffer (BioLegend) for 20 minutes at 4°C. The cells were washed with 1X Intracellular Staining Permeabilization Wash Buffer (Perm/Wash buffer, BioLegend) and incubated in 250–500 ng/mL of anti-TLRs or isotype control IgG1 mAb in 1X Perm/Wash buffer for 25 minutes at 4°C. The cells were washed with 1X Perm/Wash buffer and incubated with PE-conjugated secondary antibodies or streptavidin for 25 minutes at 4°C. The cells were washed with 1X Perm/Wash buffer and suspended in staining buffer for flow cytometry. For staining of human PBMCs, cells were incubated with Human TruStain FcX (BioLegend) for 10 min at room temperature. After the counterstaining of cell surface markers on PBMCs, the cells were subjected to intracellular staining.

### Flow cytometry analysis

Prepared samples were analyzed using an LSR Fortessa X-20 cell analyzer, FACS Aria III cell sorter (BD Biosciences), or ID7000 Spectral Cell Analyzer (SONY, Japan). Acquired data were analyzed using FlowJo software (BD Biosciences).

### Stimulation of cells

The NF-κB-GFP gene was transduced into Ramos cells as described previously (25). The cells were seeded in 96-well flat-bottom plates and stimulated with TLR ligands for 24 hours. For Ramos, the induction of GFP was detected by a flow cytometer. For human PBMCs, culture supernatant was collected, and the cytokines were detected by ELISA or AlphaLISA (PerkinElmer, Inc., Waltham, MA, USA). Antibodies were added 4 hours prior to the stimulation.

### Analysis of IgG1 production by cell-based ELISA

THP-1 cells were cultured in the complete RPMI 1640 medium (Invitrogen, Thermo Scientific, Inc.) supplemented with 10% FBS (MP biomedicals), 55 μM 2-ME (Invitrogen, Thermo Scientific, Inc.), and 1% penicillin-streptomycin (Invitrogen, Thermo Scientific, Inc.). The cells were seeded onto 96-well flat-bottom plates coated with Fibronectin (Promocell) and stimulated with Phorbol 12-Myristate 13-Acetate (FujiFilm Wako Pure Chemicals). The cells were then incubated at 37°C with 5% CO_2_ for 24 hours.

The collected culture supernatants of human purified B cells or the sequentially diluted human IgG1 (EUREKA Therapeutics, Inc., Emeryville, CA, USA), as standard, were added to the THP-1 cells in the 96-well plates. The plate was incubated at 4°C for 1 hour. After removing the culture supernatant or diluted human IgG1, the wells were washed three times with 0.05% Tween 20 in PBS (Invitrogen). The amount of human IgG1 bound to the cells was detected by horseradish peroxidase (HRP)‐conjugated secondary antibody and SureBlue Reserve TMB Microwell Peroxidase Substrate (SeraCare Life Sciences, Inc., Milford, MA, USA). The absorbance was measured using a multimode plate reader Synergy Neo2 (BioTek, VT, USA) to quantitate IgG1 concentration in the culture supernatant.

### Preparation of human PBMCs

Frozen human PBMCs were purchased from STEMCELL Technologies Inc. (Vancouver, BC, Canada) or C.T.L. (Shaker Hts, OH, USA). Frozen human purified pDCs and B cells were purchased from STEMCELL Technologies Inc. The frozen vials were kept in liquid nitrogen and thawed to use in accordance with the manufacturer’s instructions. Fresh human PBMCs were collected from healthy volunteers with informed consent. Blood was collected in EDTA-treated blood collection tubes or heparin-treated syringes and subjected to RBC lysis using RBC lysis buffer (BioLegend). Fresh human PBMCs were isolated using Ficoll-Paque PLUS (GE Healthcare) from human peripheral blood. The experiments using human samples were performed in accordance with the guidelines approved by the Office of Research Ethics, IMSUT, and Daiichi Sankyo Research Ethics Committee (Approval No. 000065).

### Analysis of cytokine production

Mouse IL-6 was measured using an Uncoated ELISA Kit (Thermo Scientific). Human IL-6 was measured using an Uncoated ELISA Kit (Thermo Scientific) or AlphaLISA (PerkinElmer). Human IFNα was measured by AlphaLISA (Perkinelmer). Mouse IL-12p40 was measured using a Duo Set ELISA kit (R&D Systems, Minneapolis, MN, USA). The absorbance was measured using a multimode plate reader Synergy Neo2 (BioTek) to quantitate the IgG1 concentration in the culture supernatant. The alpha counts were measured using a multimode plate reader, Synergy Neo2 (BioTek) to quantitate the concentration of human IL-6 and human IFNα in the culture supernatant. The other cytokines were measured using the GloMax EXPLORER (Promega) or CLARIOstar Plus (BMG LABTECH, Ortenberg, Germany).

### Antibody uptake assay

Frozen human PBMCs were prepared as described above and seeded in 96-well round-bottom plates. AF488-labeled antibodies in culture medium were added to the wells and incubated for 24 hours at 37°C. After incubation, the plates were centrifuged, and the culture medium was discarded. To quench the nonspecific fluorescence on the cell surface, cells were incubated with anti-AF488 in staining buffer (1/100 dilution) for 15 min at 4°C. Quenched cells were subjected to the counterstaining of cell surface markers, and the fluorescence of internalized AF488-labeled antibodies was detected by an ID7000 Spectral Cell Analyzer (Fig. 5G).

## Results

### Activation of the TLR7 signaling pathway in SLE

Activation of the TLR7 and TLR8 downstream signaling pathway in SLE patients was evaluated by GS analysis of whole blood samples. In the datasets of GSE88884, GSE65391, and GSE49454, SLE patients showed significantly and consistently higher scores of the TLR7 GS than healthy subjects (Fig. 1A). For the TLR8 GS scores, although a significant increase in SLE patients compared to healthy subjects was observed in GSE88884 and GSE65391 datasets, the magnitude of the median values in SLE patients was limited. Furthermore, no significant difference was observed in the GSE49454 dataset (Fig. 1A). Additionally, the TLR7 GS scores were significantly higher in the broad SLE subgroups compared to healthy subjects, regardless of races (Fig. 1B), medications (Fig. 1C), and SLE Disease Activity Index (SLEDAI) scores (Supplementary Fig. 1A). IFN GS scores showed similar profiles as TLR7 GS scores with a high correlation (*r*^2^ > 0.9, Supplementary Fig. 2). On the other hand, TLR8 GS did not correlate with SLEDAI scores (Supplementary Fig. 1B) and IFN GS scores (Supplementary Fig. 2).

### Generation of inhibitory anti-human TLR7 mAbs

In light of TLR7 activation in SLE, we undertook the development of inhibitory anti-human TLR7 (HuTLR7) mAbs. *Tlr9*^–/–^ BALB/c mice were immunized with purified HuTLR7 protein and HuTLR7-expressing Ba/F3 cells (Supplementary Fig. 3A). Anti-HuTLR7 mAbs that specifically bound to HuTLR7-expressing Ba/F3 cells but not mouse TLR7-expressing Ba/F3 cells were selected (Fig. 2A). Subsequent screening utilized the human B lymphoma cell line Ramos, in which the NF-κB-GFP reporter was transduced. The effects of these mAbs on TLR7-dependent NF-κB activation were evaluated by measuring GFP expression (Fig. 2B and C). This screening process identified inhibitory anti-HuTLR7 mAbs that dose-dependently inhibited GFP induction by the TLR7 ligand R848 (Fig. 2D). Although we treated the cells with an antibody for 4 hours prior to stimulation, co-administration of the antibodies also inhibited the TLR7 responses (Supplementary Fig. 3B).

Initial studies on species specificity revealed that clone S1, the most inhibitory clone, selectively bound to TLR7, but not to other endosomal human TLRs such as TLR3, TLR8, and TLR9 (Supplementary Fig. 3C). Clone S1 also bound to monkey TLR7, which has 97.9% amino acid sequence similarity to human TLR7 (Supplementary Fig. 3D). This cross-reactivity with monkey TLR7 is advantageous, as it allows the evaluation in monkeys. These findings demonstrate the successful development of specific inhibitory anti-human TLR7 mAbs capable of inhibiting TLR7-mediated signaling, thereby offering potential therapeutic tools for targeting TLR7 in SLE.

### Epitope mapping of inhibitory anti-HuTLR7 mAbs

To elucidate the epitope of anti-HuTLR7 mAbs, we exploited the fact that these antibodies did not bind to mouse TLR7 (Supplementary Fig. 3D). Ba/F3 cells were transduced to express chimeric mouse–human TLR7 proteins, and stained with the mAbs (Fig. 3A).

Flow cytometry analysis showed that the two inhibitory mAbs, clones S1 and S2, bound to the chimeric protein 2 (276-795), but not to the chimeric protein 3 (314-795). These data suggest that the epitopes of clones S1 and S2 contain the region 276-313. Consistently, the clones S1 and S2 did not bind to the chimeric protein 7 (1-274 and 314-795). Interestingly, the inhibitory anti-mouse TLR7 mAb clone A94B10 bounds to this chimera protein 7 (1-274 and 314-795), suggesting that both human and mouse inhibitory anti-TLR7 mAbs share the epitope.

The non-inhibitory mAb clone NF1 bound to the chimera protein 3 (314-795) but not to the chimera protein 4 (395-795). Another non-inhibitory mAb clone NF2 bound even to the chimera protein 5 (459-795). These results demonstrate that the non-inhibitory mAbs did not bind to the region 276-313.

The region 276-313 is located on the convex and upper side of the extracellular domain of TLR7 (Fig. 3B), which is distant from the ligand binding site and the dimer interface. Thus, the binding of inhibitory mAbs may not directly inhibit ligand interaction or ligand-dependent dimerization. A possible mechanism is discussed below (see the "Discussion" section).

### Inhibitory activities of the clone S1 in human PBMCs and the human TLR7 transgenic mice

The inhibitory activity of the clone S1 was investigated using *Tlr7*^‒/‒^*Rosa26*^hTLR7/hTLR7^ mice, which express human TLR7 instead of mouse TLR7 (22, 26). Using clone S1, we confirmed the expression of human TLR7 in BM-cDCs and splenocytes (Fig. 4A and Supplementary Fig. 4). BM-cDCs were treated with clone S1 for 4 hours prior to stimulation with R848 or CpG-ODN. After 24 hours, concentrations of IL-12 p40 in the supernatant were measured by ELISA. Clone S1 specifically inhibited R848-induced responses but not CpG-ODN-induced ones (Fig. 4B). *In vivo* analyses involved intraperitoneal administration of clone S1 at 25 mg/kg 1 week before administering 20 μg/mouse of R848. Peripheral blood samples were collected 6 hours after R848 administration, and serum IL-12 p40 levels were quantified by ELISA. Clone S1 significantly reduced serum IL-12 p40 levels (Fig. 4C). These results demonstrate that clone S1 effectively inhibits TLR7 responses *in vitro* and *in vivo*.

TLR7 expression in PBMCs from healthy subjects was analyzed using membrane-permeabilized staining (Fig. 4D). The highest TLR7 expression was observed in pDCs, followed by monocytes, cDCs, and B cells. TLR8 expression was detected in cDCs, monocytes, and granulocytes but not in B cells and pDCs. The inhibitory activity of clone S1 on TLR7 responses in PBMCs was further evaluated. Clone S1 at 10 μg/mL significantly inhibited IL-6 production in PBMCs stimulated with the TLR7 ligand CL264 at 500 ng/mL, but not those with the TLR9 ligand CpG-K3 at 500 nM (Fig. 4E). These findings confirm that clone S1 effectively inhibits *in vitro* TLR7 responses in PBMCs.

### Development of DS-7011a, the humanized IgG1 Ab inhibiting TLR7 responses

The cDNA sequences encoding the variable regions of the heavy and light chains of inhibitory mAbs were determined. To validate the cDNA sequences, they were expressed in Ba/F3 cells, and supernatants were used for flow cytometry analyses. For example, the reconstructed clone S1 bound to HuTLR7-expressing Ba/F3 cells (Supplementary Fig. 5A). These verified sequences of inhibitory mAbs were used to develop DS-7011a, the humanized IgG1 Ab with modification in the Fc region to reduce effector function. DS-7011a specifically bound to human TLR7, but not to TLR3, TLR8, or TLR9 (Fig. 5A). DS-7011a also bound to monkey TLR7, but not to mouse TLR7 (Fig. 5B). Additionally, DS-7011a and the clone S1 similarly bound to full-length HuTLR7 and the chimeric protein 6 (1-227 and 276-795) but not to the chimeric protein 7 (1-274 and 314-795), although their binding to the chimeric protein 6 was weaker than to full-length HuTLR7 (Fig. 5C). These data suggest that humanization does not alter the epitope of DS-7011a.

The inhibitory activity of DS-7011a was next studied. DS-7011a dose-dependently inhibited TLR7-dependent production of cytokines such as IL-6 and IFNα by human PBMCs (Fig. 5D). Consistently, TLR7-dependent IFNα production by purified human pDCs was also inhibited by DS-7011a (Fig. 5E). Finally, IgG1 production by purified human B cells was also dose-dependently inhibited by DS-7011a (Fig. 5F). These findings demonstrate that DS-7011a inhibits TLR7-mediated responses in PBMCs, pDCs, and B cells.

The internalization of DS-7011a into human PBMCs was next investigated using FACS analysis. Our previous studies indicated that anti-mouse TLR7 mAb is internalized and stays in the endosomal compartment as the immune complex (19). Human PBMCs were cultured for 24 hours with Alexa488-labeled DS-7011a and isotype control mAb. After quenching cell surface Abs, internalized Abs were detected via FACS analysis (Fig. 5G). DS-7011a was internalized to a greater extent than the isotype control Abs into TLR7-expressing cells such as B cells, pDCs, cDCs, and monocytes (Fig. 5H). In contrast, there was no difference between DS-7011a and isotype control Ab in internalization into T cells, which did not express TLR7 (Fig. 4D). These results suggest that DS-7011a is internalized in a TLR7-dependent manner and stays in the endosomal compartment in B cells, pDCs, cDCs, and monocytes in human PBMCs.

## Discussion

GS analyses indicate that TLR7 signaling is more strongly activated in SLE patients compared to TLR8 signaling. Both TLR7 and TLR8 are known to respond to RNA degradation products, specifically guanosine with oligoribonucleotides (ORNs) for TLR7 and uridine with ORNs for TLR8 (27, 28). RNA degradation is likely to generate ligands for both TLR7 and TLR8; therefore, it is unlikely that TLR7 ligands are selectively produced in SLE patients. Our flow cytometry analyses revealed that TLR7 is expressed in B cells, pDCs, cDCs, and monocytes. In contrast, TLR8 is co-expressed with TLR7 in cDCs and monocytes, but not in B cells and pDCs. This suggests that preferential TLR7 activation is due to selective activation of B cells and pDCs in SLE. RNA-associated autoantigens, such as Ro60, may be endocytosed specifically by B cells via BCRs, leading to TLR7 activation and subsequent autoantibody production (29). Once complexes of ssRNA–autoantigen–autoantibody form, pDCs can internalize these complexes via Fc receptors (FcRs) and respond to ssRNAs. The elevation of GS scores of TLR7 but not TLR8 is likely to reflect TLR7-dependent activation of B cells and pDCs.

Although cDCs and monocytes/macrophages express FcRs and can internalize immune complexes, the activation of TLR8 in these cells remains unclear. Differences between TLR7 and TLR8 have been highlighted by monogenic diseases caused by gain-of-function mutations in these genes (10, 13). For example, the Y264H mutation in TLR7 causes early-onset lupus, while TLR8 gain-of-function mutations result in recurrent infections, neutropenia, antibody deficiency, and BM failure, conditions not typical of SLE. These findings align with our results, indicating specific TLR7 activation in SLE.

DS-7011a, a humanized IgG1 Ab to TLR7, has been developed to inhibit TLR7-mediated responses in B cells and pDCs. The epitope of inhibitory mouse mAbs and DS-7011a included the region spanning position 276-313, located on the upper and convex surface of the extracellular domain of TLR7. This epitope is distant from the nucleoside-binding site and the dimer interface, suggesting that DS-7011a may not directly inhibit ligand interaction or ligand-dependent dimerization. However, this region is likely to be essential for mAb-mediated inhibition of TLR7 responses, as it is shared by the mouse inhibitory mAb A94B10, but not by non-inhibitory mAbs to human TLR7. DS-7011a may not inhibit ligand binding or dimerization, but it did inhibit the TLR7 signal. In this context, it is of note that the TLR7 antagonist does not inhibit TLR7 dimerization but alters the conformation of the TLR7 dimer (14). The TLR7 agonist induces the closed active TLR7 dimer, where the TLR7 cytoplasmic domains are close enough to activate the TLR7 signal. In contrast, the TLR7 antagonists induce the open inactive TLR7 dimer, where the cytoplasmic domains are not close enough to activate the TLR7 signal. In other words, the TLR7 agonists and antagonists stabilize the active and inactive TLR7 dimers, respectively. DS-7011a might be similar to the TLR7 antagonists.

The inhibitory mAb to mouse TLR7 is TLR7-dependently internalized (19). Consistently, DS-7011a was internalized only in TLR7-expressing cells, suggesting that the mAbs promote clustering and subsequent internalization of cell surface TLR7. It is possible that mAb-mediated TLR7 internalization alters TLR7 distribution in the endosomal compartment and that altered TLR7 distribution impairs the activation of its downstream signaling pathways. For example, TLR7 distribution and trafficking influence pro-inflammatory cytokines and type I interferon signaling (30).

Elevated TLR7 GS scores were not diminished by treatments with steroids, HCQ, and MMF, or by ethnic backgrounds, suggesting that TLR7 inhibition is effective across a broad spectrum of SLE patients, irrespective of existing therapeutic regimens and ethnic differences. Clinical trials using TLR7/8 dual antagonists, such as E6742, which binds to the antagonist-binding pocket and stabilized inactivated dimers of TLR7 and possibly TLR8, are ongoing (15, 18). However, dual inhibition of TLR7 and TLR8 may pose an increased risk of infections. TLR8 polymorphisms impairing TLR8 responses are associated with hepatitis C virus (HCV) infection (31), and TLR8 is required for neutrophil responses to SARS-CoV-2 (32). Thus, inhibition of TLR8 could potentially increase the risk of infections caused by ssRNA viruses such as HCV and SARS-CoV-2. Specific TLR7 inhibition, as achieved by DS-7011a might reduce these infection risks while providing therapeutic benefits for SLE.

In a Phase 1a study (NCT05203692), DS-7011a was generally safe and well tolerated (33). DS-7011a also dose-dependently suppressed TLR7-mediated IL-6 production, with a potent, early-onset, and long-lasting effect, indicating that DS-7011a continuously suppresses TLR7 signaling. A Phase 1b/2 study of DS-7011a in patients with SLE (NCT05638802) is currently ongoing.

## Supplementary Material

dxaf046_suppl_Supplementary_Tables_S1_Figures_S1-S5
