# Supplementary material for: Anti-human TLR7 antibody for therapeutic intervention in systemic lupus erythematosus
Source: Int Immunol. 2025 Sep 2;38(1):28–40. doi: 10.1093/intimm/dxaf046 (PMC12802918; doi:10.1093/intimm/dxaf046)
Supplement: dxaf046_suppl_Supplementary_Tables_S1_Figures_S1-S5 [file dxaf046_suppl_supplementary_tables_s1_figures_s1-s5.pdf]

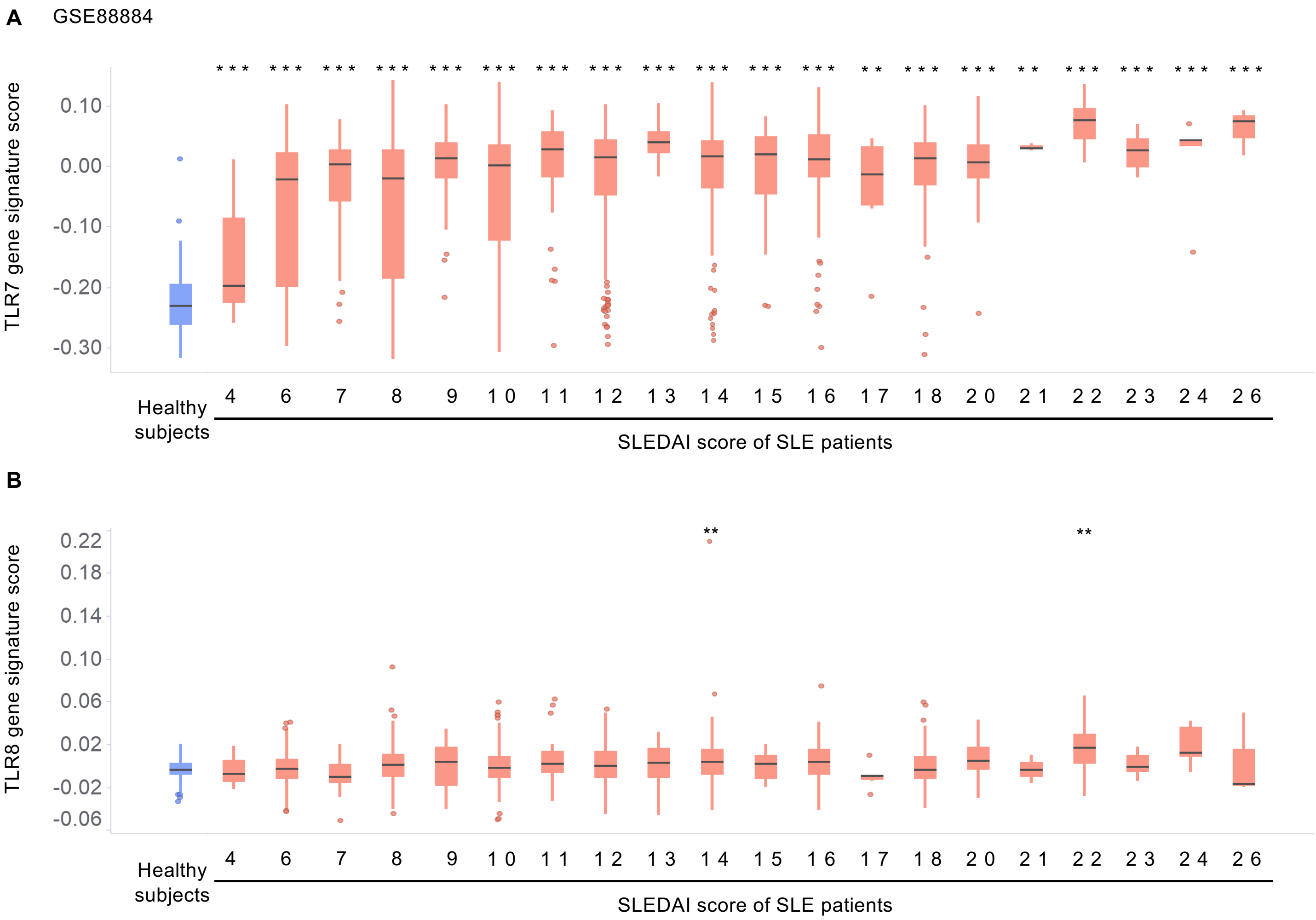

Figure S1 TLR7 gene signature scores are higher in patients with higher SLEDAI scores (A), but not for TLR8 gene signature scores (B)

TLR7 and 8 gene signature scores of SLE patients for each SLEDAI scores and healthy subjects in blood samples (GSE88884). Data were statistically analyzed by Dunnett's multiple comparison test. *: $p < 0.05$, **: $p < 0.01$, *** $p < 0.001$

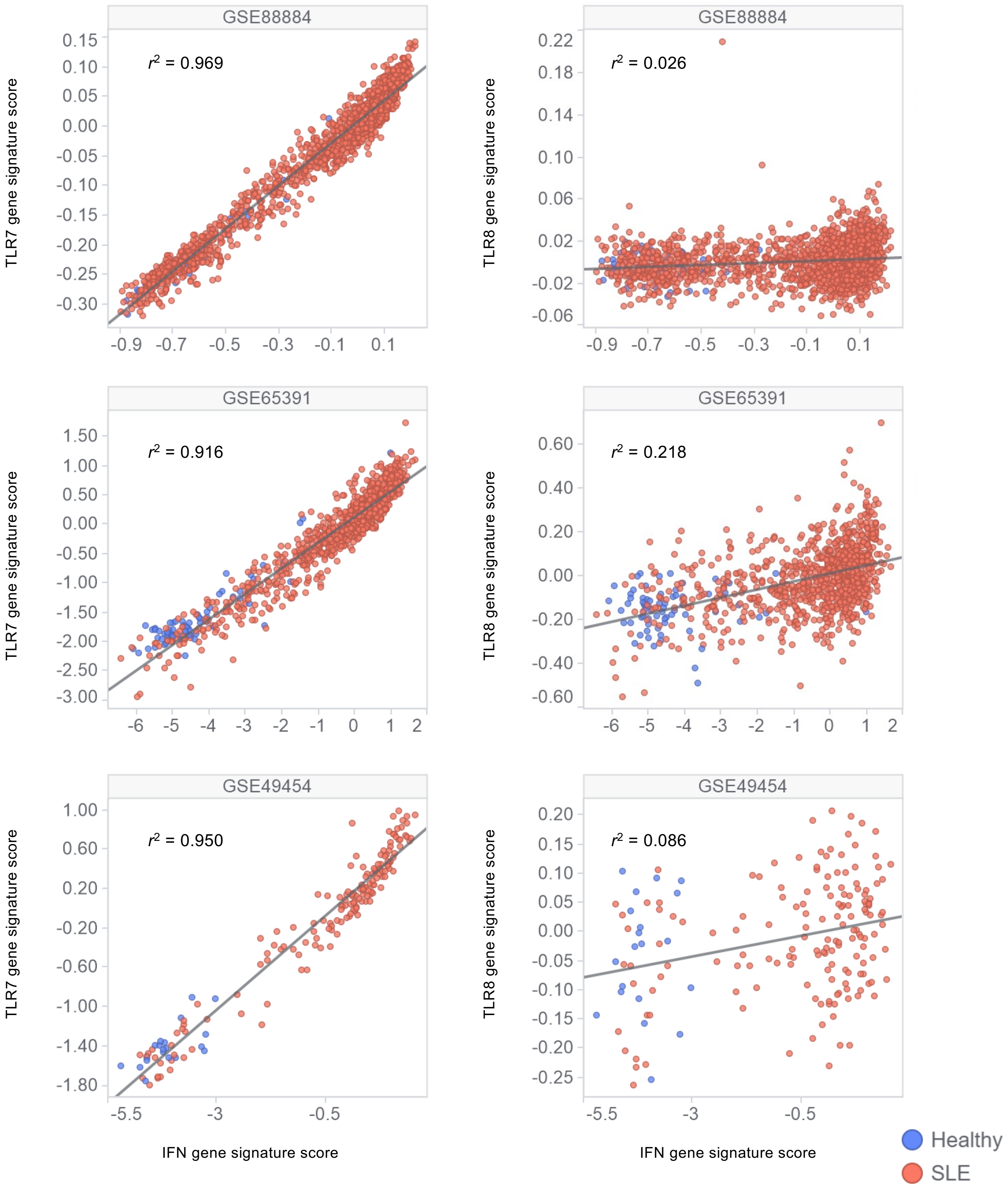

Figure S2 Relationships between Interferon (IFN) and TLR7 or TLR8 gene signature scores

The gene set of interferon gene signature was IFI27, IFI44, IFI44L and RDAS2 (Furie R, et al., Aruthritis Rheumatol. 2017;69:376-386).

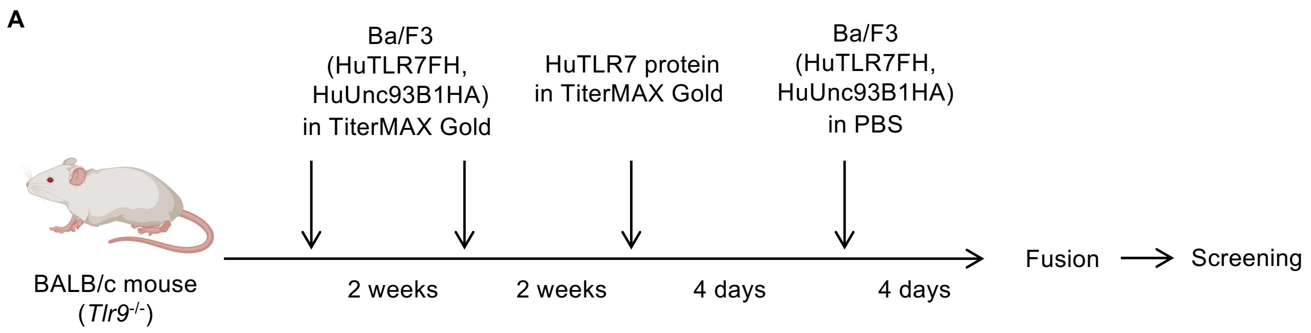

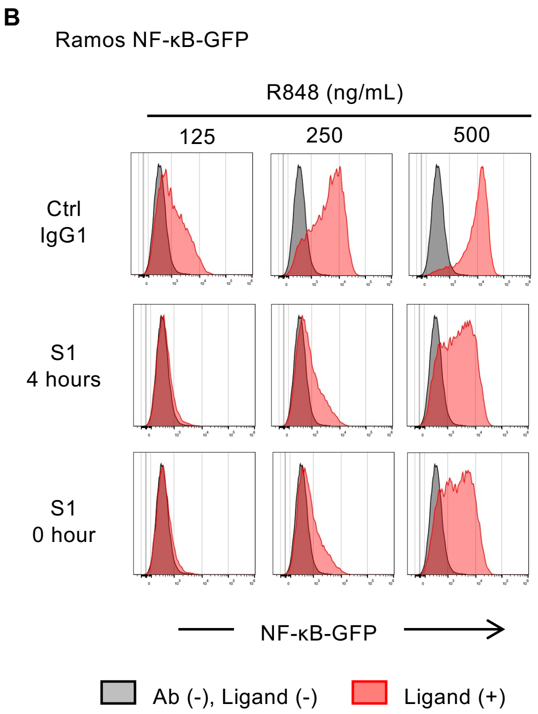

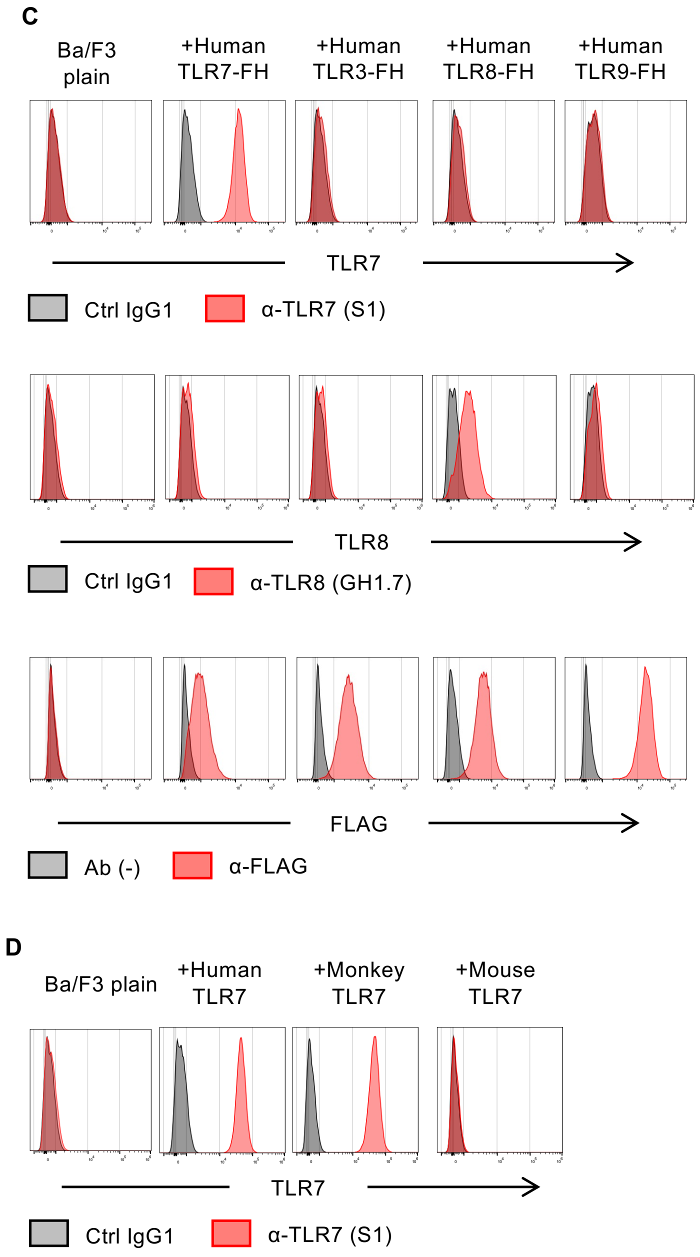

Figure S3 Generation and characterization of anti-human TLR7 mAbs

(A) Graphical method of anti-human TLR7 generation. 40-70 million of FLAG-6xHis (FH)-tagged human TLR7 (HuTLR7) and HA-tagged human Unc93B1 (HuUnc93B1)-expressing Ba/F3 cells were stimulated with 250 ng/mL of R848 for 2 hours and collected as antigen. 240 μg of purified human TLR7 (HuTLR7) protein was also used. Antigens were homogenized in TiterMAX Gold and injected subcutaneously into the tail base and foot pad. Intraperitoneal administration was also performed. 4 days before fusion, TLR7-expressing Ba/F3 cells were suspended in PBS and injected.

(B) Pre-incubation time and inhibitory effect of anti-HuTLR7. 10 μg/mL of clone S1 was incubated with Ramos NF-κB-GFP cells for indicated time. Cells were stimulated by TLR7 ligand and NF-κB-GFP induction was measured by flowcytometry.

(C and D) Internal staining of Ba/F3 cells expressing various human nucleic acid-sensing TLRs (C) or indicated species of TLR7s (D). Cells were fixed and permeabilized for intracellular staining.

At least 3 times of independent experiments were performed, and representative data were shown (B-D).

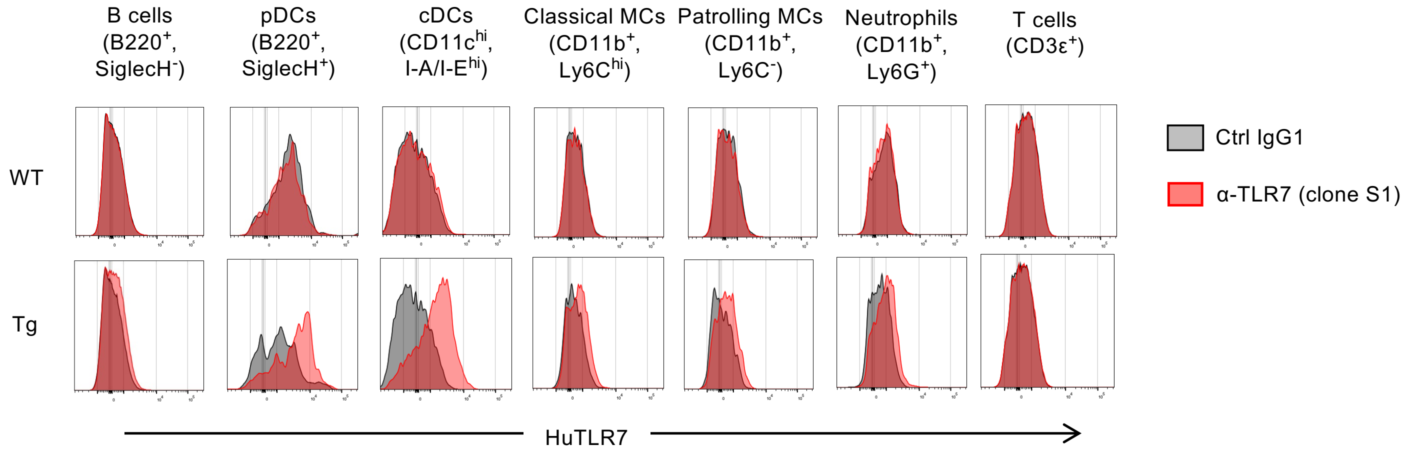

Figure S4 HuTLR7-Tg mice express antibody-detectable human TLR7

Expression pattern of human TLR7 in the splenocytes from HuTLR7-Tg mice. Cell surface markers on the splenocytes were stained for the gating of indicated populations. Intracellular TLR7 was stained using α-TLR7 (clone S1).

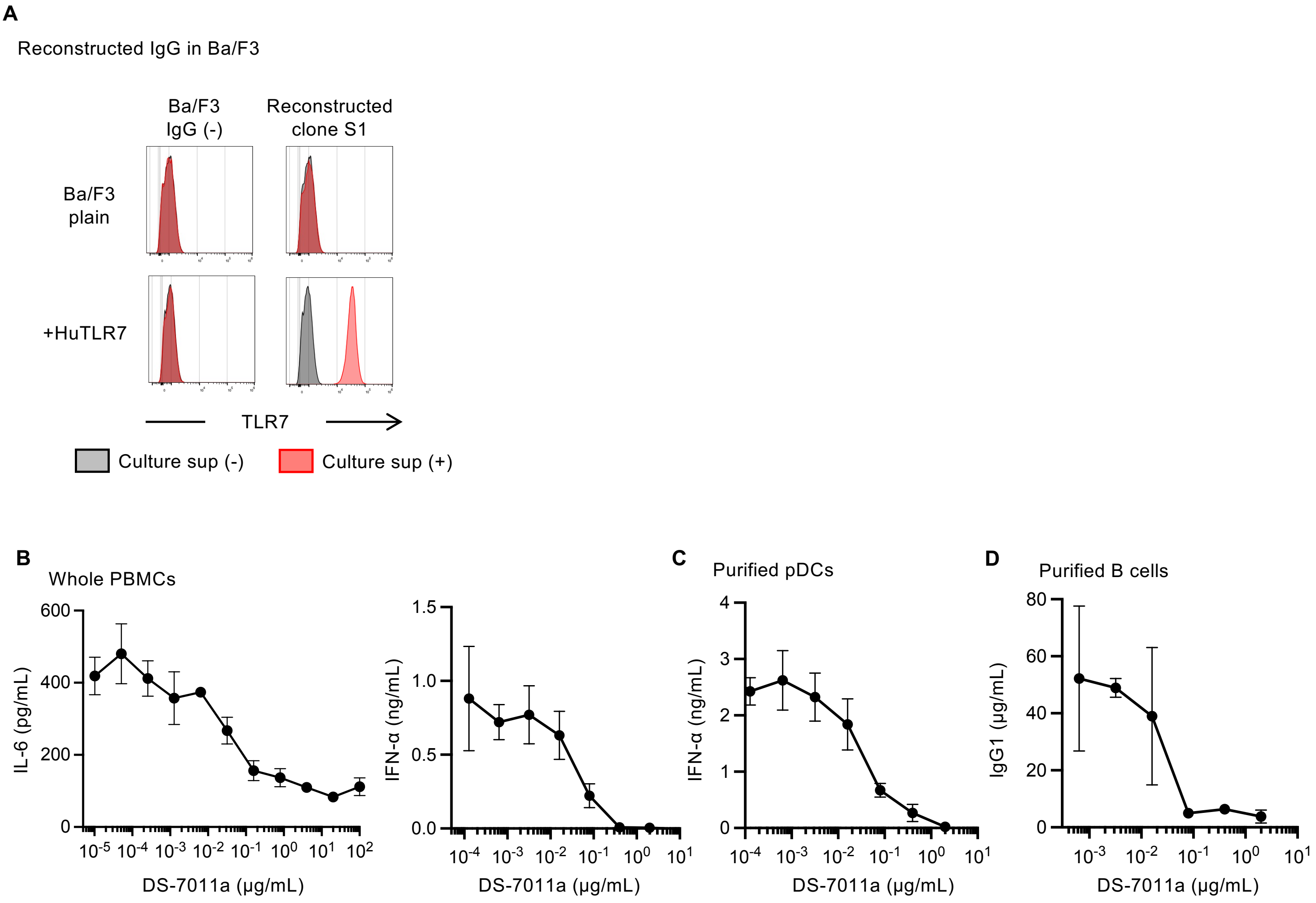

Figure S5 Reconstruction of the clone S1 and evaluation of DS-7011a

(A) The vectors coding the genes of IgG1 in anti-HuTLR7 clone S1 were transduced into Ba/F3 cells, and the supernatant was collected for the intracellular staining of Ba/F3 cells expressing HuTLR7.

(B) Inhibitory effect of DS-7011a with another donor from Figure 5D. Whole human PBMCs were incubated with antibody. After 4 hours incubation, cells were stimulated with CL264 (1 μg/mL) or ssRNA9.2s (1 μg/mL, with DOTAP) as a TLR7 ligand, respectively. Twenty hours after stimulation, culture supernatants were collected and concentrations of IL-6 and IFN-α were measured by AlphaLISA.

(C) Inhibitory effect of DS-7011a with another donor from Figure 5E. Purified pDCs were incubated with antibody. After 4 hours incubation, cells were stimulated with ssRNA9.2s (1 μg/mL, with DOTAP) as a TLR7 ligand. Twenty hours after stimulation, culture supernatants were collected and concentration of IFN-α was measured by AlphaLISA.

(D) Inhibition of TLR7-dependent IgG1 production by DS-7011a with another donor from Figure 5F. Purified B cells were incubated with DS-7011a for 4 hours before stimulation with CL264. Five days after stimulation, culture supernatants of B cells were collected and concentrations of IgG1 were measured by cell-based ELISA (D).

At least 3 times of independent experiments were performed, and representative data were shown (A-D). Wells of Alphalisa or cell-based ELISA were triplicated and the mean SD was shown (B-D).

Human TLR7 gene signature		
Gene symbol	Gene ID[a]	Relationship[b]
FN1	2335	Down
RNASE6	6039	Down
IFIH1	64135	Up
SAMD9L	219285	Up
LY6E	4061	Up
DDX60	55601	Up
XAF1	54739	Up
FFAR2	2867	Up
CCL3L3	414062	Up
EIF2AK2	5610	Up
ISG15	9639	Up
OAS2	4939	Up
SPATS2L	26010	Up
MX2	4600	Up
IFI6	2537	Up
IFIT2	3433	Up
OAS3	4940	Up
OAS1	4938	Up
RSAD2	91543	Up
IDO1	3620	Up
IFI44	10561	Up
CXCL1	2919	Up
MX1	4599	Up
CSF1	1435	Up
CMPK2	129607	Up
CCL7	6354	Up
USP18	11274	Up
CCL1	6364	Up
IFIT3	3437	Up
ITGB8	3696	Up
IRG1	730249	Up
IFI44L	10964	Up
CCL8	6355	Up
IFIT1	3434	Up
IL6	3569	Up

Human TLR8 gene signature		
Gene symbol	Gene ID[a]	Relationship[b]
TGFBI	7045	Down
PDK4	5166	Down
RNASE6	6039	Down
TREM2	54209	Down
MS4A4A	51338	Down
LRP1	4035	Down
PLBD1	79887	Down
ADORA3	140	Down
CHST13	166012	Down
CD163L1	283316	Down
WNT5A	7474	Up
TFPI2	7980	Up
IL2RA	3559	Up
G0S2	50486	Up
CCL3	6348	Up
TNFAIP6	7130	Up
ITGB8	3696	Up
CXCL8	3576	Up
TNIP3	79931	Up
CCL4	6351	Up
PTX3	5806	Up
INHBA	3624	Up
CA12	771	Up
TNF	7124	Up
PTGS2	5743	Up
CCL20	6364	Up
IL1B	3553	Up
IRG1	730249	Up
IL1A	3552	Up
IL6	3569	Up

Table S1 Gene signature definition of human TLR7 and TLR8

The gene signatures (GSs) to evaluate human TLR7 and 8 activation were defined by the top differentially expressed genes (DEGs) in the following comparisons. For TLR7 GS, human PBMCs were stimulated for 6 or 24 hours by vehicle or the TLR7 ligand CL264 at 1 μg/mL with control hIgG1 or DS-7011a. For TLR8 GS, human PBMCs or monocyte-derived macrophages were stimulated for 5 or 20 hours by vehicle or the TLR8 ligands (ssRNA40 at 1 μg/mL with DOTAP, cpd14b at 10 μM, or VTX-2337 at 1 μM). Gene expression was evaluated by microarray and RNA-seq for TLR7 and 8 GSs, respectively, and comparisons were made between vehicle and the TLR7/8 ligand or between hIgG1 and DS-7011a to select DEGs for definition of TLR7/8 GSs.

a Entrez Gene ID b Up: upregulation, Down: downregulation
